# A Population Perspective on Prevention of Dementia

**DOI:** 10.3390/jcm8060834

**Published:** 2019-06-12

**Authors:** Esmé Eggink, Eric P. Moll van Charante, Willem A. van Gool, Edo Richard

**Affiliations:** 1 Department of General Practice, Amsterdam University Medical Center, University of Amsterdam, Meibergdreef 15, 1105 AZ Amsterdam, The Netherlands; e.p.mollvancharante@amc.uva.nl; 2 Department of Neurology, Amsterdam University Medical Center, University of Amsterdam, Meibergdreef 15, 1105 AZ Amsterdam, The Netherlands; w.a.vangool@amc.uva.nl (W.A.v.G.); e.richard@amc.uva.nl (E.R.); 3 Department of Neurology, Donders Institute for Brain, Behaviour and Cognition, Radboud University Medical Center, Geert Grooteplein Zuid 10, 6525 GA Nijmegen, The Netherlands

**Keywords:** dementia prevention, Alzheimer’s disease, vascular risk factors, multi-domain interventions, public health

## Abstract

The global number of people living with dementia is expected to increase to 130 million in 2050. Based on extensive evidence from observational studies, it is estimated that about 30% of dementia cases may be attributable to potentially modifiable risk factors. This suggests that interventions targeting these factors could perhaps delay or prevent the onset of dementia. Since the vast majority of people with dementia live in low- and middle-income countries, such interventions should preferably be easy and affordable to implement across a wide range of health care systems. However, to date, results from dementia prevention trials do not provide convincing evidence that treatment of these risk factors reduces the risk of dementia. The current paper aims to give an overview of available evidence for the potential for dementia prevention. In particular, we discuss methodological issues that might complicate the development of effective prevention interventions and explore the opportunities and challenges for future dementia prevention research. Currently, several ongoing and planned trials are testing the effect of multi-domain interventions on dementia risk in high-risk populations. It is desirable that future dementia strategies also target the wider population, through interventions on the individual, community, and population level, in order to constrain the growing prevalence of dementia worldwide.

## 1. Changing Perspectives on Late-Life Dementia

The clinical picture of dementia has been recognized for centuries, but throughout time the theories on its causes have varied widely. Dementia received specific attention in 1907, when Alois Alzheimer wrote his famous case report ‘’About a peculiar disease of the cerebral cortex’’ [1]. His findings of plaques and tangles in the brain of a 51-year old patient with progressive cognitive problems were included in a leading psychiatry textbook by Emil Kraepelin, and the condition was referred to with the term ‘’Alzheimer’s disease’’ (AD) [2]. From then on, AD was considered to be a rare condition, causing dementia through plaques and tangles in relatively young people. Cognitive decline in the last decades of life, at the time referred to as senile dementia, was considered to be attributable to atherosclerosis, and stroke and was thought of as a distinct condition [3]. 

From the early seventies onwards, perceptions of the pathogenesis of senile dementia shifted from vascular mechanisms to AD pathology, based on the discovery of extensive amounts of extracellular amyloid depositions (plaques) and intracellular depositions of hyperphosphorylated tau-protein (tangles) in the brains of older people with dementia [4]. Consequently, the sharp distinction between presenile and senile dementia faded. In the early nineties, it was discovered that the specific e4 allele of Apolipoprotein E (APOE*ε*4) was associated with both early- and late-onset dementia [5,6], supporting the hypothesis that Alzheimer’s disease was the predominant cause of both early- and late-onset dementia. At this time, vascular dementia was still considered a separate, less frequent cause of dementia. 

The role of vascular pathology in the development of late-life dementia regained interest in the late nineties, when several epidemiologic and radiologic studies reported a strong relationship of cardiovascular risk factors and disease with impaired cognitive functioning [7,8]. These findings were supported by neuropathological findings. Examination of the brains of 102 elderly nuns suggested a strong interaction effect on cognitive functioning between the presence of AD pathology and lacunar strokes [9]. A large autopsy study in a population-based cohort in the United Kingdom, with a median age of 85 at death, showed that most dementia patients had a mixture of cerebrovascular and AD pathology, whereas subjects without dementia often had a considerable level of pathologies as well, or no pathologies at all [10]. Since then, numerous epidemiologic studies have investigated the relationship between vascular risk factors or vascular disease, and stroke development, and late life dementia [11,12,13]. Based on several more recent studies, it is perceived that the presence and mutual interaction of genetic factors, such as carrying the APOE*ε*4 allele, and vascular factors are involved in the development of multiple brain pathologies, including amyloid plaques, tangles containing hyperphosphorylated tau, and different vascular lesions [14,15,16]. These brain pathologies all increase the likelihood to develop mild cognitive impairment (MCI) and dementia [17], but they are not sufficient to fully explain either onset, course, or specific clinical symptoms. 

## 2. Exploring the Window of Opportunity for Dementia Prevention

The concept of dementia caused by multifaceted brain disease implies a wide range of possible strategies for dementia prevention and treatment. The need for such strategies is emphasized by the large number of people living with dementia worldwide, which is expected to rise from 47 million in 2015 to over 130 million in 2050, largely due to the increasing life expectancy [18]. It is estimated that 90% of dementia patients are older than 75 years, and 75% are older than 80 years of age [19]. Strategies to prevent dementia among people without the disease could perhaps delay its onset and reduce the prevalence of dementia [20]. Since it is expected that by 2050 68% of all people with dementia live in low- and middle-income countries (LMIC) [18], such strategies should ideally be easy and inexpensive to implement on a large scale across a wide variety of health care systems. 

Observational studies suggest a number of modifiable factors that are associated with dementia risk and could serve as a target for prevention. Elevated blood pressure, body mass index (BMI), elevated total cholesterol levels [21,22,23,24,25,26], diabetes mellitus [27], current smoking [28], depression [29], physical inactivity [30], cognitive inactivity [31], poor diet [32], and low educational attainment [33] are well-established factors that are independently associated with an increased risk of dementia. Even small improvements of the modifiable dementia risk factors on the individual level have the potential to lead to a substantial reduction of dementia cases at the population level, due to the high global prevalence of these risk factors [34]. By calculating population-attributable risks for seven well-established dementia risk factors (diabetes mellitus, midlife hypertension, midlife obesity, physical inactivity, depression, smoking, and low educational attainment), and taking inter-relatedness into account, it was estimated that 30% of all dementia cases worldwide can be attributed to these potentially modifiable risk factors [35], with low educational attainment, smoking, and physical inactivity carrying the strongest risk. This suggests a large window of opportunity for dementia prevention. 

The high prevalence of these modifiable factors raises the question of whether population-based prevention strategies could reduce the prevalence of dementia. Over the years, many community programs have been designed to reduce cardiovascular disease (CVD) risk. Controlled before–after studies have shown that, in general, these programs can be effective at improving cardiovascular risk factors and, in some cases, reducing incident CVD and mortality [36]. Although risk factors are largely similar for CVD and dementia, no comparable studies have been performed to study the effect of community prevention programs on cognitive functioning or dementia. However, five large studies have compared dementia occurrence between two time points in well-defined geographical areas. Four of five studies showed a slight reduction of dementia prevalence, which could potentially be attributed to population-level investments, including improved education and better prevention and treatment of vascular conditions [37]. 

## 3. Dementia Prevention Trials

In the last two decades, several intervention studies have been performed to test the hypothesis that dementia can be delayed or prevented by improving individual risk factors or the overall dementia risk profile in people free from cognitive impairment at baseline. We distinguish single-domain interventions, targeting a single risk factor, and multi-domain interventions, targeting multiple dementia risk factors simultaneously. Below, we will discuss these studies with dementia as a primary or secondary outcome. 

### 3.1. Single-Domain Interventions

Although the list of potential interventions is very long [38], we will restrict our overview to the interventions for which most robust evidence from clinical trials and meta-analyses is available. As such, we do not intend to be exhaustive here.

Treatment of hypertension may reduce the risk of dementia via blood pressure lowering mechanisms, but also through other, perhaps antihypertensive class-specific, effects [21,39,40,41]. Results of hypertension trials have been encouraging, but are still inconclusive. A meta-analysis of four placebo-controlled trials of antihypertensive treatment with incident dementia as a primary outcome showed a combined risk ratio of 0.87 (95% CI 0.76 to 1.00; *N* = 16,595 individuals; *n* = 786 dementia cases), favoring treatment [42]. A more recent meta-analysis included nine blood pressure-lowering trials, including two lifestyle interventions, with a median follow-up of 3.9 years. The pooled risk ratio for incident dementia was 0.93 (95% CI 0.84 to 1.02; *N* = 57,682; *n* = 2131 dementia cases) [43]. The recently published Systolic Blood Pressure Intervention Trial: Memory and Cognition in Decreased Hypertension sub-study (SPRINT-MIND) assessed whether intensive blood pressure treatment with any agent, aiming for levels lower than 120 mmHg, could reduce incident dementia compared with standard blood pressure control, aiming for levels lower than 140 mmHg, in over 9000 patients (50+) with hypertension. The trial was ended prematurely because of beneficial effects on cardiovascular events and all-cause mortality in the intervention group. Pre-planned secondary analyses showed no significant effect on probable dementia (HR 0.83; CI 0.67 to 1.04; *N* = 8563; *n* = 325 dementia cases), but a significant reduction of incident MCI (HR 0.81; CI 0.69 to 0.95; *N* = 8563; *n* = 640 probable MCI cases) after a median intervention period of 3.3 years and a median follow-up period of 5.1 years [44]. Taken together, despite promising results from observational studies [21], these two meta-analyses and recent RCT failed to provide convincing evidence that dementia can be delayed or prevented with blood pressure treatment, but point estimates consistently suggest a potential preventive effect. 

Type 2 diabetes mellitus (T2DM) may increase dementia risk through different mechanisms including cerebrovascular damage, insulin resistance, and mitochondrial dysfunction [45,46]. A recent systematic review identified seven randomized controlled trials to assess the effects of different T2DM treatment strategies on cognitive function and incident dementia [47]. Three studies were included in the efficacy analyses and used cognitive function or incident dementia as outcome measure. All three studies were at unclear risk of bias. Two of these studies compared intensive glycemic control versus standard glycemic control [48,49]. There was no significant difference between the two groups with regard to the number of participants who declined by at least 3 points on the mini-mental state examination (MMSE) over five years (RR 0.98; CI 0.88 to 1.08; *N* = 11,140 individuals; 1 study), incident dementia (RR 1.27; CI 0.87 to 1.85; *N* = 11,140 individuals; *n* = 109 dementia cases; 1 study) [49], or MMSE score after 40 months (MD −0.01; CI −0.18 to 0.16; *N* = 2794 individuals; 1 study) [48]. The third study compared glibenclamide with repaglinide. After 12 months, a small advantage of glibenclamide on MMSE score was found (MD −0.90; CI −1.68 to −0.12; *N* = 156 individuals; 1 study) [50]. 

Despite observational evidence [23,24], to date no trials have shown beneficial effects of cholesterol-lowering treatment on dementia risk. A systematic review identified two RCTs that compared the effect of a statin versus placebo on cognitive decline and incident dementia among individuals with increased cardiovascular risk. Both studies had a low risk of bias. No difference was found with regard to incident dementia (OR 1.00; CI 0.61 to 1.65; *N* = 20,536; *n* = 62 dementia cases; 1 study) between simvastatin and placebo. No effect of simvastatin or pravastatin was found on cognitive function, assessed by five different cognitive tests [51]. According to current guidelines, a very high percentage of participants between 40 and 75 years old are eligible for statin prescription, with the aim to prevent cardiovascular disease [52]. Although the prevention of stroke can be expected to lower the risk of dementia, there is no direct evidence for this effect so far.

Physical activity is thought to decrease dementia risk through multiple mechanisms, including increased neurogenesis, angiogenesis, and synaptic plasticity and anti-inflammatory effects [53]. Moreover, physical activity can have beneficial effects on other factors that are associated with dementia risk, including obesity, dyslipidemia, and high blood pressure. A recent systematic review investigated 32 trials with a follow-up of more than 6 months, to assess the effectiveness of physical activity interventions on cognitive function among adults without a diagnosis of cognitive impairment. Included studies targeting only physical activity involved aerobic training (six studies, 531 individuals), resistance training (three trials, 315 individuals), and tai chi (one trial, 93 individuals). Evidence from these trials was insufficient to draw any conclusion about a beneficial effect on cognitive function [54]. Because of the beneficial effects of physical activity on obesity and the risk of CVD, public health campaigns and public health initiatives to facilitate physical activity are widely applied. To date, whether this will reduce the risk of dementia remains uncertain.

### 3.2. Multi-Domain Interventions

Exposure to a combination of modifiable dementia risk factors may have a synergistic effect on risk of cognitive decline and dementia [55,56]. Therefore, multi-domain interventions, targeting more than one risk factor, may be a more appropriate approach to study dementia prevention. In the past decade, several multi-domain trials have been performed, testing varying interventions across a wide range of sample sizes and follow-up times. We will discuss the main multi-domain intervention studies in terms of sample size and follow-up time with dementia, MCI, or cognitive decline as primary end-point (Table 1). 

The Dutch prevention of Dementia by Intensive Vascular Care (preDIVA) [57] cluster-randomized trial compared the effect of a 6-year, intensive, nurse-led multi-domain cardiovascular care intervention with usual care on the cumulative incidence of dementia and disability. 116 General practices were randomly assigned to one of the conditions. 3526 individuals without dementia, aged 70–78 years, participated. After a median follow-up of 6.7 years, primary outcome data were obtained in more than 98% of the participants. No significant effect was found of the intensive cardiovascular care on incident dementia (HR 0.92; CI 0.71 to 1.19; *N* = 3454 individuals; *n* = 233 dementia cases) and disability. 

The Finnish Geriatric Intervention Study to Prevent Cognitive Impairment and Disability (FINGER) [58] compared the effect of a multi-domain intervention, including nutritional guidance, physical activity, cognitive training, and monitoring of modifiable dementia risk factors, with general health advice (control group) on cognitive function, assessed with an extensive neuropsychological test battery (NTB). 1260 Individuals without dementia, aged 60–77 years, with an increased dementia risk in terms of 6 or more points on the Cardiovascular Risk Factors, Aging and Dementia (CAIDE) risk score, were randomly assigned to either of the treatment arms. After two years, the intervention group showed a slightly larger improvement on the standardized NTB compared with the control group (between-group difference in change score per year 0.022; CI 0.002 to 0.042; *N* = 1190 individuals). 

The French Multidomain Alzheimer Preventive Trial (MAPT) [59] studied the effects of omega 3 polyunsaturated fatty acids and the effect of a multi-domain intervention, consisting of group sessions targeting cognitive training, physical activity, and nutrition on cognitive function. Participants were eligible when they were 70 years or older and either had subjective memory complaints, limitations in one instrumental activity of daily living, or slow walking speed. 1680 Participants were randomly assigned to one of four groups: the multi-domain intervention combined with omega 3 polyunsaturated fatty acids, the multi-domain intervention with placebo, and omega 3 polyunsaturated fatty acids with no other intervention or placebo alone. After three years, there were no significant differences in cognitive function, assessed with a composite score of four cognitive tests, between any of the treatment groups and the placebo alone group: between-group differences were 0.093 (95% CI 0.001 to 0.184; *N* = 1525 individuals) for combined intervention, 0.079 (95% CI −0.012 to 0.170; *N* = 1525 individuals) for the multi-domain intervention plus placebo group, and 0.011 (95% CI −0.081 to 0.103; *N* = 1525 individuals) for the omega 3 polyunsaturated fatty acids group. 

## 4. Explaining the Gap between Observational and Interventional Studies

A substantial gap exists between the results from many observational studies, suggesting optimism, and the rather sobering results from dementia prevention trials. Hence, it could be that vascular factors have an association, rather than a causal relationship, with dementia risk. However, most of Hill’s criteria for causation, such as consistency and plausibility [60], are met. Although the current evidence does not support a protective effect of preventive interventions for dementia, particularly for hypertension, there is a rather consistent signal in the direction of a preventive effect. Moreover, it is conceivable that methodological issues, which have been associated with the design of dementia prevention trials [61,62,63], lead to type II errors, masking “true” effects of multi-domain interventions, and causing apparent inconsistency with observational evidence. 

### 4.1. Age of the Target Population and J-Shaped Curves

An important issue when designing a dementia prevention trial is the optimal age range of the target population. A target population that is too young would require infeasible follow-up periods or sample sizes, due to the low incidence of dementia in younger age. Conversely, a target population that is too old would probably lead to decreased efficacy of the intervention, because the relationship between some risk factors and dementia becomes more complex with age [63]. The association between blood pressure during late-life and dementia is suggested to follow a U- or J-shaped curve, with both high and low values imposing increased dementia risk [64]. This is consistent with ample research on the relationship between blood pressure and cardiovascular disease [65]. With regard to BMI, a similar J-shaped relation with dementia risk is suggested in late-life, with elevated BMI levels being associated with lower, and being underweight with increased, dementia risk [66], suggesting a similar type of J-shaped curve as with blood pressure. Likewise, high total serum cholesterol concentrations in late-life have been associated with decreased dementia risk [24,67]. It is unclear when the directions of these associations change. Nevertheless, it is conceivable that the target populations from the three multi-domain interventions described above, with age-ranges 60–77, 70+, and 70–78 years, respectively, were too old to benefit from the interventions. These complex relationships pose a major challenge for future dementia prevention trials. Clearly, one size does not fit all, but with regard to age it is currently unclear what the optimal target values for blood pressure, BMI, and cholesterol might be. 

### 4.2. Risk Profile of the Target Population

The level of quality and accessibility of standard preventive care that is available for the target population affects the degree of contrast a trial may yield. Subgroup analyses of the preDIVA study show the strongest effects of the intervention in participants with untreated hypertension and in participants without history of cardiovascular disease [57]. It could well be that an effect of the intervention was not found in the three multi-domain intervention trials, because high-quality cardiovascular risk management was already available for both intervention and control participants. As such, future studies may need to target populations at high risk who lack access to high-quality preventive health care. Policymakers and health organisations alike may need to actively target those persons that are typically not represented in clinical trials, but are at highest risk. 

### 4.3. Hawthorne and Treatment Effects in the Control Condition

Another challenge is the observed improvement on primary and secondary outcomes of the control group in some multi-domain intervention studies [57,58]. This is illustrated by the decrease in blood pressure in both study arms of the preDIVA trial. The mean difference in systolic blood pressure between baseline and follow-up was 8.3 mmHg in the intervention group and 4.6 mmHg in the control group, suggesting initiation of treatment by a general practitioner or specialist or changes in lifestyle behaviour by the participant following the baseline measurements. Additionally, changed behaviour of participants or healthcare professionals as a reaction to the awareness of the study (Hawthorne effect) is likely to play a role [68]. Both mechanisms could mask the “true” contrast between the intervention and control condition, leading to type II errors. 

### 4.4. Competing Risk of Death

Age is the most important risk factor for dementia. Starting at the age of 60, the incidence of dementia doubles with every 6.3 years increase in age [18]. It is likely that, due to shared risk factors, dementia prevention trials have beneficial effects on cardiovascular endpoints, and, as a consequence, on mortality. Therefore, effective multifactorial interventions could paradoxically increase dementia incidence rates when death is delayed. If not taken into account, this could lead to serious underestimation of the effectiveness of dementia prevention interventions.

## 5. Future Directions

### 5.1. Strategies to Deal with Limited Statistical Power

When designing dementia prevention trials, sufficiently large sample sizes and/or long follow-up periods are paramount to reach statistical power, due to the time lag between the optimal timing of the intervention and dementia onset. Hence, given these preconditions, funding dementia prevention trials will remain a daunting challenge. 

One potential approach towards longer follow-up is open label extension of studies, as was done in the Syst-Eur trial [13]. However, selective attrition will be a complicating factor for such observational extensions. Another strategy to overcome lack of power is to collaborate with other (international) research groups, enabling the design of multi-national trials and pooling of data of previous trials where possible and appropriate. An example is the European Dementia Prevention Initiative (EDPI) consortium, a collaboration of five European institutes, including the three research groups involved in the FINGER, MAPT, and preDIVA trials, respectively [69]. A third strategy could involve selection of a primary outcome that is likely to emerge earlier in life than dementia onset. Examples are cognitive impairment, existing dementia risk scores, or biomarkers presumed to reflect biological processes eventually leading to dementia. However, the uncertain association between biomarkers and cognition renders this a suboptimal primary outcome with regard to clinical relevance. A fourth solution could be to exclusively target individuals with an increased dementia risk who are still free from cognitive impairments. Numerous strategies exist to estimate dementia risk, including the use of biomarkers, imaging [70], family history [71], and dementia risk scores [72,73]. Obviously, from a population perspective, the use of (invasive) biomarkers is not feasible, certainly not in LMIC, but simple and readily available risk markers such as a positive family history or the presence of multiple dementia risk factors can be applied on a large scale at low cost. Some researchers have also used signs of cognitive decline to indicate high dementia risk. However, the latter approach is accompanied by a relatively high risk of including individuals with an early stage of dementia, in whom the intervention is less likely to be effective [74]. A fifth approach could be to target populations with poor access to preventive healthcare quality, such as in LMIC. These populations could be a promising target for lifestyle interventions, since the incidence of dementia is relatively high and the peak incidence is at younger age than in high-income countries (HIC) [18]. Moreover, the prevalence of dementia risk factors in these countries is higher than in HIC [75]. 

### 5.2. Ongoing and Planned Multi-Domain Dementia Prevention Trials

For successful implementation in LMIC, dementia prevention interventions should ideally be easily available, accessible, and affordable. These criteria are often met by web-based interventions, such as electronic health (eHealth) and mobile health (mHealth), especially because the majority of the world population uses internet these days and in countries with limited internet access it is increasing rapidly [76]. Four currently ongoing or planned multi-domain interventions will be testing the effectiveness of such digital dementia prevention interventions (Table 2). 

The ongoing multi-national Healthy Aging Through Internet Counselling in the Elderly (HATICE) trial, performed by the EDPI consortium, is comparing a coach-supported, interactive internet platform, stimulating self-management of cardiovascular risk factors, with a sham platform without interactive features, for 18 months. The study population consists of approximately 2724 individuals, aged 65 years or older, and with an increased cardiovascular risk. The primary endpoint is a composite cardiovascular risk score, including systolic blood pressure, low-density-lipoprotein, and BMI. Cognitive function is a secondary outcome [77].

An ongoing cluster-randomized trial in Thailand with 3600 participants is comparing a three-year digital, coach-supported lifestyle modification intervention on four domains (diet, physical activity, alcohol drinking, and smoking) with care as usual. Participants are eligible when they are between 45 and 75 years of age and do not have a diagnosis of dementia, chronic kidney disease, diabetes, chronic obstructive pulmonary disease, cancer, or CVD. The primary outcome, measured after ten years, is incident dementia. Cognitive function, assessed with the MMSE, is one of the secondary outcomes [78]. 

The Maintain Your Brain (MYB) trial is comparing a digital platform with interactive modules on physical activity, diet, mental health, and cognitive training with a digital platform containing static information about dementia risk factors. The study population will consist of approximately 8500 individuals, recruited through an existing Australian cohort of non-demented community dwelling individuals aged between 55 and 77 years. The primary outcome, measured after three years, is cognitive change on a composite score of cognitive functioning. Secondary outcomes are incident dementia and change in dementia risk [79]. 

The planned Prevention Of Dementia Through Mobile Phone Applications (PRODEMOS) trial, initiated by the EDPI consortium, takes place in the United Kingdom (UK) and in Beijing, China [80]. A total of 2400 individuals, aged 55–75 years, with an increased dementia risk profile, and of low socioeconomic status in the UK, are randomized between a coach supported, interactive smartphone application, stimulating self-management of dementia risk factors; and a sham application without interactive features. The primary endpoint, measured after 18 months, is the CAIDE dementia risk score. 

World Wide Fingers is an interdisciplinary network that arose from the FINGER trial. The multi-domain lifestyle intervention showed a modest beneficial effect on cognitive function after two years in a Finnish geriatric population. The same intervention is going to be tested in the United States, in rural China, in Singapore, and in several European countries [81]. 

### 5.3. Population-Based Approaches

Most of the ongoing trials are testing individual interventions in specific high-risk populations. However, the majority of dementia cases occur in individuals with low or intermediate risk [82]. It is therefore desirable that future dementia prevention strategies also target the wider population. Interventions targeting (a subgroup of) the population as a whole require different strategies. In addition to the individual level, primary prevention can be delivered at the community or the population level. Public health interventions that target common risk factors, such as discouraging smoking and encouraging a healthier lifestyle, can be implemented at several levels, and may include media campaigns, legislative changes, and preventive measures in working spaces and the community. Evaluating the effects of such interventions is complex, and may require different approaches than the classical parallel group randomised controlled trial. In addition to alternative methodologies to evaluate effectiveness, measures related to implementation will have to be taken into account, and such studies may require alternative large-scale governmental funding. Since risk factors for dementia largely overlap with risk factors for CVD, implementation in existing healthcare would probably benefit from an integrated approach, targeting dementia, CVD, and other non-communicable diseases [83].

## 6. Conclusions

Although results from observational studies suggest optimism, to date, results from dementia-prevention trials do not provide convincing evidence that treatment of these risk factors reduces the risk of dementia. However, some interventions, especially in intensive hypertension management, appear promising in the reduction of dementia risk and cognitive decline. Taking into account that the majority of dementia cases occur in LMIC, interventions should be easy and affordable to implement. Currently, several ongoing trials are testing the effectiveness of eHealth and mHealth interventions in high-risk individuals. Further implementation research on broadly available preventive interventions in the general population is warranted, to achieve global impact on dementia prevalence. 

## Figures and Tables

**Table 1:** Multi-domain dementia prevention trials.

	preDIVA	FINGER	MAPT
Sample size	3526	1260	1680
Age range	70–78	60–77	70+
Main inclusion criteria	Not demented ^b^	Dementia risk score ≥6 ^a^ Cognitive performance at mean or slightly lower level	Not demented ^b^ Memory complaints or limitations in daily living or slow gait speed
Intervention	Nurse-led intensive vascular care	Diet advice, exercise, cognitive training and vascular care	Cognitive training, advice on physical activity and nutrition, and vascular care +/− omega 3 polyunsaturated fatty acids
Intervention period	6–8 years	2 years	3 years
Follow-up period	6–8 years	2 years	3 years
Primary outcome	Dementia, disability ^d^	Cognitive function ^c^	Composite *z*-score of 4 cognitive tests ^e^
Main secondary outcomes	Cardiovascular disease, vascular factors, cognitive decline, depression	Vascular and lifestyle factors, depressive symptoms, disability	Physical performance, depression

FINGER: Finnish Geriatric Intervention Study to Prevent Cognitive Impairment and Disability; MAPT: Multidomain Alzheimer Prevention Study; preDIVA: prevention of Dementia by Intensive Vascular Care. ^a^ assessed with Cardiovascular Risk Factors, Aging, and Dementia (CAIDE) risk score; ^b^ defined as no clinical diagnosis and a Mini-Mental State Examination >23; ^c^ assessed with the neuropsychological test battery (NTB); ^d^ assessed with the AMC Linear Disability Score; ^e^ items from the Free and Cued Selective Reminding test, Mini-Mental State Examination, Digit Symbol Substitution Test, and Category Naming Test.

**Table 2:** Planned and ongoing multi-domain dementia prevention trials.

	HATICE	Impact of Lifestyle Modification on Prevention of Dementia	MYB	PRODEMOS
Start of recruitment	March 2015	March 2016	May 2018	January 2020
Sample size	2724	3600	8500	2400
Recruiting countries	The Netherlands, Finland, France	Thailand	Australia	United Kingdom, China
Age range	65+	45–75	55–77	55–75
Main inclusion criteria	Not demented ^a^, ≥2 cardiovascular risk factors	Thai nationality, no diagnosis of dementia, diabetes, COPD, cancer, or CVD	No diagnosis of dementia or severe depression	Not demented ^a^, ≥2 dementia risk factors
Intervention	Coach-supported Internet platform for self-management of cardiovascular risk factors	Coach-supported computer program on diet, physical activity, alcohol drinking, and smoking	Digital modules on physical activity, nutrition, peace of mind, and brain training	Coach-supported smartphone app for self-management of dementia risk factors
Intervention period	1.5 years	3 years	3 years	1.5 years
Follow-up period	1.5 years	10 years	3 years	1.5 years
Primary outcome	Composite *z*-score of SBP, LDL cholesterol, and BMI	Incident dementia	Global cognition composite domain score ^b^	CAIDE score, implementation outcomes
Main secondary outcomes	Individual factors from composite score, incident CVD	Incident T2DM, CVD, cancer, COPD, mortality	Incident dementia, dementia risk	Individual components of CAIDE score, disability, cost-effectiveness

BMI: body mass index; CAIDE: Cardiovascular Risk Factors, Aging, and Dementia risk score; COPD: chronic obstructive pulmonary disease; CVD: cardiovascular disease; HATICE: Healthy Aging Through Internet Counselling in the Elderly; Impact of Lifestyle Modification on Prevention of Dementia: Impact of Lifestyle Modification on Prevention of Dementia, Chronic Kidney Disease, Diabetes, Chronic Obstructive Pulmonary Disease, Cancers, and Cardiovascular Disease in a Thai General Population: Cluster Randomized Controlled Trial; LDL: low-density lipoprotein; MYB: Maintain Your Brain; PRODEMOS: Prevention Of Dementia Through Mobile Phone Applications; SBP: systolic blood pressure; T2DM: type 2 diabetes mellitus. ^a^ defined as Mini-Mental State Examination >23; ^b^ Maintain Your Brain Battery.
